# Effect of inter-dental abutment distance on the impression accuracy of digital and conventional methods

**DOI:** 10.25122/jml-2023-0103

**Published:** 2023-05

**Authors:** Shouka Shalileh, Kamyar Abbasi, Hamed Azhmand, Seyed Ahmad Ghoraishian, Mina Mohaghegh

**Affiliations:** 1. Department of Prosthodontics, School of Dentistry, Shiraz University of Medical Sciences, Shiraz, Iran; 2. Department of Prosthodontics, School of Dentistry, Shahid Beheshti University of Medical Sciences, Tehran, Iran; 3. School of Dentistry, Shiraz University of Medical Sciences, Shiraz, Iran

**Keywords:** impression, dental abutment, accuracy, digital impression, conventional impression, intraoral scanner

## Abstract

This study aimed to examine the effect of inter-dental abutment distance on the accuracy of digital and conventional impression methods. Five maxillary and mandibular models were prepared with different inter-dental abutment distances. Digital scans were obtained using an extraoral laboratory scanner as reference data. Each group was scanned 8 times using the intra-oral scanner for the digital method. For the conventional impression method, 8 additional silicone impression material was used to generate the stone casts from each group. Then casts were scanned. In the next step, stereolithography (STL) data was exported from the scans. The STL files were super-imposed on the reference scans using 3shape dental designer software to make the measurement. Kolmogorov-Smirnoff was used to determine if the data were normally distributed. In the digital impression method, as the abutment distance increased, the accuracy decreased. Various inter-dental abutment distances in digital groups showed significant differences (p=0.016) in impression accuracy, while the difference among conventional groups was not statistically significant (p=0.822). In the digital method, the mean inter-dental abutment between the 4-5 and 3-7 groups, 4-6 and 3-7 groups had a significant difference (p<0.05). However, the conventional method revealed no significant differences (p>0.05) between groups. In conclusion, when the inter-dental abutment distance exists and is surrounded by soft tissue, the possibility of error in the digital impression method is higher than in the conventional impression method.

## INTRODUCTION

The goal of a dental impression is to accurately replicate a patient's intraoral state and transform it into a tangible model. A precise impression is a basis for successful treatment in all prosthetic restorations [1]. Several factors can affect impression accuracy, including impression technique and materials vacuum versus hand mixing, water/powder ratio, type of impression tray, setting time, accurate cast preparation, and finally, obtaining a suitable framework. Lack of accurate impression can lead to biomechanical complications and marginal bone loss [2-9]. For many years, conventional impression methods were the only methods available. While the accuracy of these impression materials cannot be dismissed, the conventional method undeniably presented several shortcomings. For instance, dental restorations created using these conventional impressions were prone to human errors, and various external factors could negatively impact the accuracy of this impression technique [10-15]. This quest for maximum accuracy and fidelity led to the development of the oral digital scanning system in response to the popularity of the conventional impression method [16, 17].

The mid-1980s marked the introduction of digital impressions and scanning systems in dentistry. In recent years, with the improvement of digitalization, digital impression-taking, and CAD/CAM have become practical and feasible alternatives to conventional methods for impressions. Digitally digitizing the gypsum cast and fabricating a three-dimensional (3D) digital model for restoration design is the process behind this technology. CAD-CAM technology can produce a virtual 3D model with digital intraoral scanners [18, 19]. Digital impressions have many advantages, including rapid casting, storing information indefinitely, 3D pre-visualization of tooth preparation, and transfer of digital scans between the dental office and the laboratory [20-24]. This method reduces many common errors related to the conventional method, including dimensional variations of impression materials, dental stone expansion, and human errors [25,26]. Digital scanners can overcome errors such as the presence of material on the teeth. Conventional and digital impressions can be affected by the interdental abutment space, which is a critical factor [27], and the entire dental arch, including mucosal and soft tissue areas. However, no previous report has provided a detailed assessment of the accuracy of the conventional method and digital scan data based on the inter-dental abutment distance in the impression process [1]. The effect of inter-dental abutment distance on impression accuracy should be clarified to promote the clinical application of dental casts for semi-edentulous patients with different types of defects. Therefore, this study evaluated digital and conventional impression accuracy in relation to inter-dental abutment distance.

## MATERIAL AND METHODS

### Model teeth preparation

**Figure 1.**
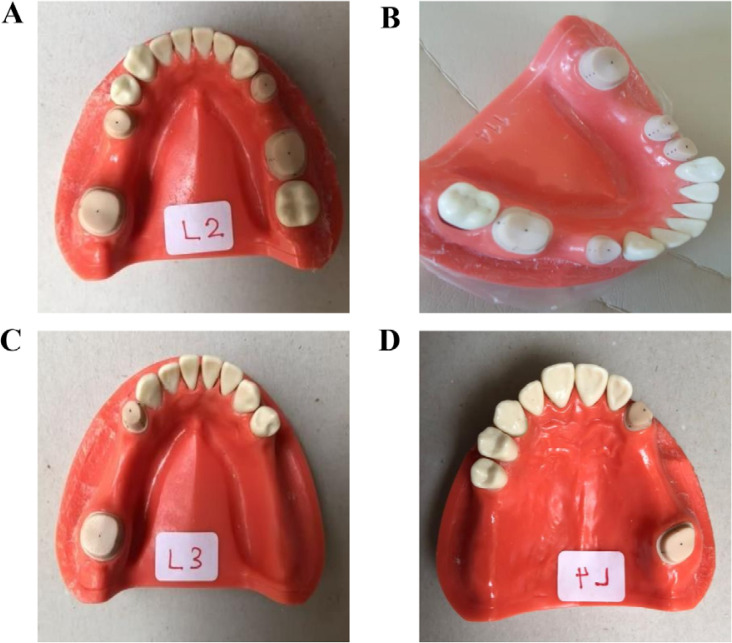
Typodonts prepared for the study with different inter-dental abutment distances; A: L1, B: L2, C: L3, D: L4

Five maxillary and mandibular models were prepared with different inter-dental abutment distances. The first one had a complete set of teeth, and the first and second premolars were prepared (4-5). While the first molar and premolar were prepared, the second one missed the second premolar (4-6). The third one missed the first molar with the second premolar, and the second molar was prepared (5-7). The fourth one missed the second premolar and first molar, with the first premolar and second molar (equal three-size premolar) being prepared (4-7). The fifth one missed the first, second premolar, and first molar (equal four-size premolar), with the canine and second molar being prepared (3-7) (Figure 1 A-D). The typodont teeth were scanned digitally with TRIOS intraoral scanner (3shape Copenhagen, Denmark). In accordance with the instructions provided by the manufacturer, five digital impressions were taken. Intra-oral scans were transferred to the software and designed with a supragingival chamfer margin and 10-degree taper using CAD/CAM machine (dental designer-3 shape-trios, Denmark 2019). Prepared abutment teeth were printed from resin with a dental printer (Asiga, 2019, Australia) and placed on the dentiform model. Four references were marked on the abutment's lingual, buccal, mesial, distal, and occlusal surfaces. The dentiform models were scanned with a laboratory scanner (3 shape-D810, 2019, Denmark) and saved as a reference (control) scan.

### Conventional impressions

For conventional impressions, we used perforated plastic stock trays. Our materials were heavy and light in viscosity. Using heavy body (ISO 4823 type 0) and light body (ISO 4823 type 3) techniques, a single operator with more than 3 years of experience made 8 impressions with additional silicone material from each group after applying tray adhesive. A 10-minute disinfection period was applied to every impression. Impression plaster was poured over impressions after being stored for 8 hours. At ambient temperature and humidity, the stone cast was stored for 48 hours after removing the impression trays. Threeshape D810, 2019, Denmark, was used to scan the casts. Next, stereolithography (STL) data was exported from the scans. Using 3shape dental-designer software, the STL files were superimposed on the reference scans.

### Digital impression

A single operator with over 3 years of experience with intraoral scanning began to capture 8 scans from each group with the intraoral scanner. The data was imported into 3shape-dental designer software. 3shape dental designer software was used to super-impose the impressions after receiving all STL datasets. The super-imposition and measurements were done, and data were collected for further analysis (Table 1).

**Table 1.** Workflow of the digital impression system

System	Surface conditioning	Scanning principle	Scan procedure	STL export
Trios	None	Confocal laser, Continues image	According to the manufacturer's instruction	Direct via 3shape communicate portal

### Statistical analysis

Statistical analyses were performed using IBM SPSS Statistics for Windows, version 24. The dataset was imported from an Excel spreadsheet, and the Kolmogorov-Smirnov test was used to determine the normality of data distribution. Levene's test was utilized to verify the equality of variances among all test groups (α=0.05). To identify statistical discrepancies among the groups, a one-way ANOVA was conducted. Furthermore, the post hoc LSD test was employed to evaluate variations in inter-dental abutment distances within each group.

## RESULTS

### The mean difference of inter-dental abutment distance between two scanning methods

According to the Kolmogorov-Smirnov test, deviations within each group were normally distributed. Based on the Levene test, no equality of variance was found (p=0/05). The ANOVA test revealed statistically significant differences in precision across the various groups. These statistical findings are described in Table 2. The impression precision of all groups was determined by measuring the distance difference between the reference scan and the study groups. The mean distance difference (mm) is shown in Table 3.

**Table 2.** The mean difference of inter-dental abutment distance between two scanning methods across study groups

Digital method						
Groups	Number	Mean (mm)	Standard deviation	Std error	Minimum	Maximum
4-5	8	0.01225	0.006756	0.002389	0.002	0.025
4-6	8	0.01600	0.007464	0.002639	0.007	0.030
4-7	8	0.02650	0.012728	0.004500	0.012	0.049
5-7	8	0.03688	0.035470	0.012541	0.008	0.106
3-7	8	0.04825	0.031450	0.011119	0.020	0.105
Total	40	0.02798	0.025135	0.003974	0.02	0.106
**Conventional method**						
**Groups**	**Number**	**Mean (mm)**	**Standard**	**deviation**	**Std error**	**Minimum**	**Maximum**
4-5	8	0.03825	0.0220327	0.07791	0.014	0.081
4-6	8	0.05363	0.027039	0.09560	0.017	0.106
4-7	8	0.04575	0.009677	0.03421	0.028	0.056
5-7	8	0.04150	0.044049	0.15574	0.003	0.115
3-7	8	0.04075	0.024400	0.08627	0.016	0.076
Total	40	0.04398	0.026836	0.04243	0.003	0.115

**Table 3.** Detailed analysis of distance differences between two scanning approaches across study groups

Digital method					
Comparison	Sum of squares	Df	Mean square	F	Sig.
Between groups	0.007	4	0.002	3.518	0.016
Within groups	0.018	35	0.001		
Total	0.025	39	-		
**Conventional method**					
**Comparison**	**Sum of squares**	**Df**	**Mean square**	**F**	**Sig.**
Between groups	0.01	4	0.000	0.379	0.822
Within groups	0.027	35	0.001		
Total	0.028	39	-		

### Significance of distance differences between the two scanning methods across the study groups

The ANOVA analysis in Table 3 demonstrates the distance difference between the two scans across the test groups. Accordingly, digital groups showed a significant difference (p=0.016), while the difference among conventional groups was not statistically significant (p=0.822).

### Multiple groups comparison of inter-dental abutment distance

Additionally, in this study, we evaluated the inter-dental abutment distance differences in each group using the post-hoc-LSD test. The multiple comparison results revealed significant differences in impression accuracy within the digital impression method. Specifically, there were statistically significant differences (p<0.05) in the impression accuracy for the inter-dental abutment distances between the 4-5 and 3-7 groups, as well as between the 4-6 and 3-7 groups (Table 4). In contrast, the conventional impression method did not show any significant differences in mean inter-dental abutment distances between the groups (p>0.05) (Table 5).

**Table 4.** Multiple groups comparison of inter-dental abutment distance in digital impression method

Digital impression method				
I group	J Group	Mean difference (I-J)	STD. error	Sig
4-5	4-6	-0.003750	0.011204	0.740
	4-7	-0.014250	0.011204	0.212
	5-7	-0.024625	0.011204	0.035
	3-7	-0.036000	0.011204	0.003
4-6	4-5	0.003750	0.011204	0.740
	4-7	-0.010500	0.011204	0.355
	5-7	-0.020875	0.011204	0.071
	3-7	-0.032250	0.011204	0.007
4-7	4-5	0.014250	0.011204	0.212
	4-6	0.010500	0.011204	0.355
	5-7	-0.010375	0.011204	0.361
	3-7	-0.021750	0.011204	0.060
5-7	4-5	0.024625	0.011204	0.035
	4-6	0.020875	0.011204	0.071
	4-7	0.010375	0.011204	0.361
	3-7	-0.011375	0.011204	0.317
3-7	4-5	0.036000	0.011204	0.003
	4-6	0.032250	0.011204	0.007
	4-7	0.021750	0.011204	0.060
	5-7	0.011375	0.011204	0.317

**Table 5.** Multiple groups comparison of inter-dental abutment distance in conventional impression method

Conventional impression method				
I group	J Group	Mean difference (I-J)	STD. error	Sig
4-5	4-6	-0.015375	0.014031	0.281
	4-7	-0.010000	0.014031	0.481
	5-7	-0.003250	0.014031	0.818
	3-7	-0.002500	0.014031	0.860
4-6	4-5	0.015375	0.014031	0.281
	4-7	0.005375	0.014031	0.704
	5-7	0.012125	0.014031	0.393
	3-7	0.012875	0.014031	0.365
4-7	4-5	0.010000	0.014031	0.481
	4-6	0.005375	0.014031	0.704
	5-7	0.006750	0.014031	0.633
	3-7	0.007500	0.014031	0.596
5-7	4-5	0.003250	0.014031	0.818
	4-6	0.012125	0.014031	0.393
	4-7	0.006750	0.014031	0.633
	3-7	0.000750	0.014031	0.958
3-7	4-5	0.002500	0.014031	0.860
	4-6	0.012875	0.014031	0.365
	4-7	0.007500	0.014031	0.596
	5-7	0.000750	0.014031	0.958

## DISCUSSION

The application of digital technology in prosthetic treatment has witnessed a notable rise in recent years. Particularly, the clinical utilization of digital impressions acquired through intraoral scanners has become increasingly prevalent in various prosthetic procedures [28]. Dental clinicians have long been concerned about achieving accurate impressions with high precision [29]. Today, digital intraoral scanners have gained prominence due to their superior accuracy compared to conventional impression methods. However, the accuracy and outcomes of both conventional and digital impressions are influenced by various factors [19]. This study aimed to evaluate the effect of inter-dental abutment distance on impression accuracy with the digital method using TRIOS 3 intraoral scanner and the conventional impression method using additional silicone material. In our study, the accuracy of both digital (12±6μm-48±31μm) and conventional (38±22μm -53±27μm) methods was within clinically acceptable ranges (10-70μm) [30]. According to studies, an increased distance between the abutment tooth and the scanning origin can lead to localized data distortion and decreased linear accuracy [31]. However, in this study, with increasing inter-abutment distance, both methods were found to be more accurate. To make a suitable restoration, the preparation of the tooth must be accurately recorded during the impression process. Obtaining acceptable restorations requires impression materials that are stable and dimensionally accurate [32-35]. Currently, additional silicone material is the gold standard material for impression-making. It is widely used because of its high accuracy, good dimensional stability, good elastic properties, high tear strength, excellent recovery from deformation on removal, and short working and setting time [36]. According to some studies, using the proper type of scanner plays an essential role in digital impression accuracy. The impression accuracy in the digital method depends on two parameters: the resolution of the scanning and the algorithm's accuracy. TRIOS-3 scanners were used in this study. The third generation of TRIOS is a convenient solution for impression that performs three tasks simultaneously: intraoral scanning for fast, 3D and color impression, and intraoral camera.

Consequently, digital impressions offer the advantage of archiving all documents [37]. Jong-Eun *et al*. have highlighted the potential benefits of using artificial landmarks in edentulous areas, and they have also noted that the choice of the intraoral scanner can influence the quality of scans. In their study, they evaluated the Cerec Omnicam by Sirona, the CS3500 scanner by Carestream, and the Trios scanner by 3shape [38]. However, in our study, we specifically used the Trios intraoral scanner, and our samples were dental bridges, not edentulous areas. Therefore, the use of aluminum landmarks may not be necessary in our context.

Impression accuracy is a parameter that indicates the precision of the intra-oral scanning method [39]. Some studies have shown that the conventional impression method can provide higher accuracy than the digital method for specific reasons [19]. In contrast, digital systems display scanned teeth in a magnified form on a monitor and can re-scan areas that were not properly scanned. This minimizes the possibility of impression errors [40]. Many intraoral scanners worldwide differ in many factors, including the type of cameras used, the image capture process, and the type of digital models created [41]. One of the factors that can affect the accuracy of impression is the presence of soft tissue due to the inter-dental abutment.

Also, in this study, we demonstrated that not only is inter-dental abutment distance an effective factor in impression accuracy, but increasing this distance can also lead to an increase in accuracy errors, especially in the digital intra-oral scanner. As with the effect of the inter-dental abutment distance on digital and conventional impression accuracy, its impact on digital and conventional impressions has been studied comparatively in many studies [42, 43]. Based on the results of our study, the inter-dental abutment distance difference between the two impression methods was significant. Tan *et al*., consistent with our study, showed that reducing the dental abutment distance increased the digital impression accuracy. In contrast, this parameter did not affect the conventional impression technique [44]. Thanasrisuebwong *et al*. showed that impression accuracy and precision errors increased with the increasing inter-dental spaces [45]. In addition, Parkan *et al*. demonstrated a significant difference between inter-implant distances scanned by two types of scanners (TRIOS and CEREC) [45]. Unlike the present study, Basaki *et al*. showed that the distance between the tooth models does not affect impression accuracy [46]. The results showed that the largest average of inter-dental abutment discrepancy between two scans in the digital method was observed in the 3-7 group with the highest error rate of 105 micrometers, which was much higher than the standard error rate (10-70 micrometers) [30].

In addition, according to the results of the current study, in the digital impression method, the impression accuracy for the inter-dental abutment distance of the 4-5 and 3-7 groups, and 4-6 and 3-7 groups had a significant difference (p<0.05) when the maximum difference in inter-dental abutment distance was observed. Accordingly, the conventional impression method showed better results when the inter-dental abutment distance increased. Kim *et al*. [47] showed that the conventional scanning method is more accurate than the digital one. Also, Ender *et al*. emphasized the simultaneous use of conventional and digital scanning methods [48]. Basaki *et al*. compared impression accuracy in conventional and digital methods in another study. The results of this study, consistent with our study, showed that the three-dimensional difference between the impression model and the reference model in the digital method was less than in the digital method [46]. On the other hand, Abdu *et al*., inconsistent with our study, showed that digital impressions are more accurate than conventional impressions [30]. Furthermore, Alikhasi *et al*. demonstrated that digital molding is more accurate than conventional molding [17]. Accordingly, Berkman *et al*., inconsistent with our study, demonstrated no significant difference in the distance deviations between tooth models [49]. One of the factors that can cause contradictory results is the difference in the impression accuracy measurement method. In this study, a 3-shape-trios scanner was used to scan the templates, and the 3-shape-D810 scanner was used as a reference. However, Papaspyridakos *et al*. scanned all stone impressions with a 6 μ precision scanner (IScan D103i; Imetric) as references, and the STL data sets were used for comparison with digital impression data [50].

There are some limitations due to factors such as saliva, blood, or patient movement, which can increase the rate of errors. Moreover, scans were done on acrylic teeth, representing different optical characteristics than natural teeth. In addition, only one type of intraoral scanner was assessed in this survey, so it is suggested that future studies evaluate the accuracy of other types of intraoral scanners used in clinical settings. Accordingly, more studies are suggested to support the results of this study.

## CONCLUSION

The accuracy of both digital and conventional methods was within clinically acceptable ranges. When the inter-dental abutment distance exists and is surrounded by soft tissue, the possibility of error in the digital impression is higher than in the conventional impression. The greater inter-dental abutment distance can disrupt the digital scan and decrease the digital accuracy, while it does not significantly affect the accuracy of the conventional method.
